# Determinants of gestational syphilis among women attending prenatal care programs in the Brazilian Amazon

**DOI:** 10.3389/fpubh.2022.930150

**Published:** 2022-11-09

**Authors:** Thais Lopes do Amaral Uchôa, Eliete da Cunha Araújo, Richardson Augusto Rosendo da Silva, Rubenilson Valois, Wanderson Santiago de Azevedo Junior, Valéria Gabriele Caldas Nascimento, Cintia Yolette Urbano Pauxis Aben-Athar, Andressa Tavares Parente, Eliã Pinheiro Botelho, Glenda Roberta Oliveira Naiff Ferreira

**Affiliations:** ^1^Program of Post-Graduation in Nursing, Federal University of Pará, Belém, Brazil; ^2^Program of Post-Graduation in Collective Health, Federal University of Rio Grande do Norte, Rio Grande do Norte, Brazil; ^3^Program of Post-Graduation in Nursing, State University of Pará, Belém, Brazil

**Keywords:** congenital syphilis, case-control studies, pregnant women, syphilis, antenatal

## Abstract

**Background:**

There was a high proportion of pregnant women who were attending prenatal care who were not tested for syphilis or tested but not treated, among priority countries. The coverage for prenatal care visits, syphilis screening, and treatment are priority indicators for monitoring of the elimination of syphilis. The aim was to determine the factors associated with gestational syphilis among postpartum women who were in a prenatal care program in the Brazilian Amazon.

**Methods:**

An unmatched case–control study was conducted at the hospital in Brazil. Data collection was carried out from November 2020 to July 2021 during hospitalization using a pretested structured questionnaire. The criteria for selection of cases and control followed the guidelines established by the Ministry of Health of Brazil; postpartum women with a laboratory diagnosis based on treponemal and/or nontreponemal tests, symptoms of syphilis or asymptomatic, treatment or not treated, and in a prenatal care program. Gestational syphilis cases were identified as women who tested positive for syphilis, and those who tested negative were controls, at minimally one prenatal care visit, childbirth, and/or the puerperium. The sample size encompassed 59 cases and 118 controls (1: 2 ratio of cases to controls). Data were analyzed using Minitab 20^®^ and BioEstat 5.3^®^ software. The odds ratio was calculated by multiple logistic regression.

**Results:**

One hundred and seventy-seven postpartum women were included in the study, 59 cases and 118 controls. Among all participants, 95.5% (169) were tested for syphilis in any trimester during pregnancy and at the delivery and 4.5% (8) were tested in the maternity only, at the time childbirth and/or puerperium. The final multiple logistic regression model evidenced that cases had higher odds compared to controls if they had past history of sexually transmitted infections (AOR: 55.4; *p:* 0.00), difficulty talking about condom use with their sexual partner (AOR: 4.92; *p:* 0.01), one to six prenatal care visits (AOR: 4.93; *p:* 0.01), had not received a sexually transmitted infections test result in the maternity hospital (AOR: 4.09; *p:* 0.04), lower monthly income (AOR: 4.32; *p:* 0.04), or one to three miscarriages (AOR: 4.34; *p:* 0.01).

**Conclusion:**

The sociodemographic, programmatic, obstetric, and sexual factors are associated with gestational syphilis among postpartum women.

## Introduction

The elimination of mother-to-child transmission (EMTCT) of *Treponema pallidum* is a public health priority (1, 2). It is estimated that congenital syphilis (CS) rates decreased globally between 2012 and 2016, from 539 to 473 per 100,000 live births (1) suggesting an expansion of prenatal healthcare (PNC) services. However, there are still regional disparities (1–3).

In Brazil, between 2012 and 2019, the congenital syphilis and gestational syphilis (GS) rates presented an increased trend by 112.5 and 282.46%, respectively (3). Gestational syphilis poses a burden particularly in populations affected by socioeconomic inequality, like in the Brazilian Amazon. In this region, the GS and CS rates per 1,000 live births were 17.5 and 7.1 in 2019, respectively (3, 4). Additionally, a recent study mentioned the existence of gaps mainly in the early diagnosis and adequate treatment of gestational syphilis (4).

The Brazilian government has allocated resources to the EMTCT through the expansion of coverage for primary healthcare, rapid tests, and treatment for syphilis with autonomy in prescribing benzathine penicillin by nurses (5–7). However, the data reported suggest that these efforts have not been sufficient (3, 4).

Previous studies have reported that risk factors for GS are: one to three PNC visits (8), less education, a history of abortion (9, 10), previous sexually transmitted infections (8, 10, 11), multiple sex partners (9–11), unmarried status, travel of sex partner in the past 12 months (10), substance use (11). However, only one case-control study has assessed programmatic (access and quality of prenatal care) factors (8).

There has been no study to identify programmatic factors, sociodemographic, obstetric, and behavioral factors associated with GS among postpartum women with access to PNC in areas affected by social inequality (8–11). Therefore, identifying whether the access to a PNC program and an adequate number of PNC visits are capable of mitigating these factors is important for preventing mother-to-child transmission of syphilis (12–14).

This study aimed to determine the factors associated with GS among postpartum women who were part of PNC programs in the Brazilian Amazon.

## Materials and methods

### Study design

This observational, unmatched case-control study design was conducted at a referral hospital in Belém city, the capital of Pará state in northern Brazil. Among Brazilian states and capitals, Pará and Belém are the sixth and third lowest on the municipal Human Development Index (Pará = 0.746; Belém = 0.698), respectively. In addition, both this state and capital have low family health strategy coverage rates of 57.6 and 23.1%, respectively (15, 16). The study hospital was chosen because it is a public referral hospital in Belém, which provides the highest level of childbirth and PNC high risk for pregnant women in the state. Data collection was conducted from November 2020 to July 2021.

All participants of this study were postpartum women who had delivered at the study hospital and received PNC exclusively by the primary care. All women who were unable to answer the questionnaire were excluded.

The results of both the rapid test for syphilis (treponemal test) and Venereal Disease Research Laboratory (VDRL) nontreponemal test, as well as syphilis treatment data, were collected directly from the patients' medical records and the prenatal card. In this study, we followed the Brazilian Ministry of Health criteria for GS (17) which are defined as a case meeting the following conditions: (a) asymptomatic for syphilis, who during prenatal care, childbirth, and/or puerperium presents with at least one reagent test (treponemal or nontreponemal with any titration) and without any record of previous treatment; (b) symptomatic for syphilis, who during PNC, childbirth, and/or puerperium presents with at least one reactive test (treponemal or nontreponemal with any titration); or (c) during PNC, childbirth, and/or the puerperium, presents with a reactive nontreponemal test with any titration and reactive treponemal test, regardless of syphilis symptoms and prior treatment.

As a control population, we included postpartum women with nonreactive treponemal or nontreponemal tests during PNC, childbirth, and/or puerperium. In addition, women were excluded if the newborn's syphilis test (VDRL) was positive or clinical signs were exhibited.

The sample size was calculated using Epi Info™ version 7.2.2.16, StatCalc – Sample size and power function for unmatched case-control. The parameters used were: 95% Confidence Interval, 80% power, 13.7% the proportion of control exposed (less than three prenatal care visits) (8), and an odds ratio of 3.6. The final sample size was 47 cases and 94 controls (1:2 ratio of case to control) after subtracting the 25% non-response rate from the original 59 cases and 118 controls.

### Data sources

Data were collected by trained nurses during the hospitalization in face-to-face interviews using a pretested, structured questionnaire, based on the medical records, the prenatal cards, and previous studies (8, 18–20).

The following variables were collected from the prenatal cards, medical records and interviews: (a) Sociodemographic factors — date of birth (current age - years), education level, marital status, monthly income, occupation, access to the internet; (b) Programmatic factors — number of visits for prenatal care, gestational age, syphilis screening, difficulty performing rapid tests for syphilis, difficulty talking about condom use with sexual partner, received prenatal card in the first visit for prenatal care, prenatal card in the maternity hospital, sexually transmitted infections test result in the maternity hospital; (c) Obstetrics and sexual factors — condom use, past history of sexually transmitted infections, age at first sexual intercourse (years), number of miscarriages, gravidity, and parity. A draft instrument was sent to the nurse researchers to check the content for clarity and adequacy. After these revisions, a pretest was conducted at the hospital using a 5% random sample of the total sample size. Women included in the pretest did not participate in the final sample. After the pretest, there were further questionnaire modifications, but no tests were performed to assess the reliability and validity of the questionnaire.

### Variables

The principal outcome variable was the syphilis status of the women during prenatal care, childbirth, and/or puerperium. Participants were divided into two groups: reactive treponemal or nontreponemal test results (case) and not reactive (control). This variable was treated as a binary dependent variable. The response event selected was a case (reactive results). The independent variables were sociodemographic factors and programmatic, obstetric, and sexual aspects.

### Statistical analysis

Data were analyzed using Minitab 20^®^ and Biostat 5.3^®^ software. Categorical variables were expressed as percentages, and numerical variables were expressed as the mean and standard deviation (SD). An odds ratio (OR), calculated by multiple logistic regression, was used to determine the factors associated with GS among postpartum women who attended a PNC program.

A bivariate regression analysis was used to assess the association between each independent variable and the dependent variable. All variables with a *p* < 0.20 were entered into a multiple logistic regression equation using a stepwise approach. Multicollinearity was verified using the variance inflation factor (VIF); the correlations were tolerable with a VIF < 5. A crude OR and adjusted odds ratio (AOR) with their respective 95% CIs were used to evaluate the effect. All *p-*values < 0.05 were considered statistically significant. To interpret the results, quality tests, the coefficient value of the independent variable, *Z*-value, and 95% CI and OR were considered in the regression.

## Results

A total of 177 postpartum women (59 cases and 118 controls) comprised the sample of the study. Of the 177 women, only 31.5% were tested for syphilis during the first or third trimesters, or at childbirth, as recommended by the Brazilian Ministry of Health. Among the two groups, 20.3% of the case group and 36.4% of the controls received this test as recommended (Table 1).

**Table 1:** Sociodemographic factors associated with gestational syphilis, in the Brazilian Amazon, 2020–2021.

**Variable**	**Case**	**Control**	**Total**	**Regression**		
	***n* (%)**	***n* (%)**	***n* (%)**	***p***	**OR**	**95% IC**
**Current age (years)**						
15-24	31 (52.5)	52 (44.1)	83 (46.9)	0.64	1.30	0.44; 3.75
25-34	22 (37.3)	53 (44.9)	75 (42.4)	0.85	0.90	0.30; 2.66
35-41	6 (10.7)	13 (11)	19 (10.7)	1		
Mean (SD)	24.8 (6.18)	26.1 (6.17)	25.7 (6.18)			
**Education level**						
Elementary	17 (28.8)	27 (22.9)	44 (24.9)	0.39	0.73	0.36; 1.48
High school/University	42 (71.2)	91 (77.1)	133 (75.1)	1		
**Marital status**						
Single	17 (28.8)	30 (25.4)	47 (26.6)	0.24	1.76	0.67; 4.59
Stable union	33 (55.9)	60 (50.9)	93 (52.4)	0.22	1.71	0.72; 4.05
Married	9 (15.3)	28 (23.7)	37 (20.9)	1		
Monthly income (minimal wage)^*^						
Equal or less than 1 minimum wage	53 (89.8)	78 (66.1)	131 (74.0)	0.00	4.52	1.79; 11.4
More than 1 minimum wage	6 (10.2)	40 (33.9)	46 (26)	1		
**Current job**						
Housewife/No	37 (62.7)	52 (44.1)	89 (50.3)	0.02	2.13	1.12; 4.05
Yes	22 (37.3)	66 (55.9)	88 (49.7)	1		
**Access to the internet**						
No	2 (3.4)	8 (6.8)	10 (5.7)	0.36	0.48	0.09; 2.34
Yes	57 (96.6)	110 (93.2)	167 (94.3)	1		

* Brazilian monthly minimum wage in November 2021–1,090 Real per month.

All women in the case group were treated during pregnancy, childbirth, or puerperium. The therapeutic schemes are described below: 78% (46) benzathine benzylpenicillin 2.4 million international units administered in three-doses; 1.7% (01) benzathine benzylpenicillin 2.4 million international units administered as a single-dose; 3.4% (02) doxycycline 100 mg in postpartum women due to penicillin allergy (reactive result occurred at delivery or puerperium) and 18.6% (11) had no details of the therapeutic scheme or number of doses. In the case group, 84.7% (*n* = 50) had newborns diagnosed with CS. Among all participants, 95.5% (169) were tested for syphilis in any trimester during pregnancy and at the delivery and 4.5% (8) were tested in the maternity only, at the time childbirth, and/or puerperium (data not shown in tables).

Table 1 shows the sociodemographic characteristics of the two groups. Among all women, the mean age was 25.7, SD: ±6.18 years (95% CI: 24.7; 26.6; age range: 15 to 41 years). The age distributions were similar among cases (Mean: 24.8, SD: 6.18) and controls (Mean: 26.1, SD: 6.17). The odds of having a lower income are 5 times greater in the case group (OR = 4.52; 95% CI: 1.79–11.4; *p* = 0.00). In addition, the odds of being unemployed were two times greater in the case group than in the control group (OR: 2.13; 95% CI: 1.12; 4.05 *p* = 0.02). There were no statistically significant differences in education level, marital status, or internet access between the case and control groups (Table 1).

The programmatic aspects (access, offer, and use of healthcare services) for the control and case groups are shown in Table 2. The number of PNC visits was higher for the controls (Mean: 7.1; SD: 2.7) than for the case group (Mean: 5.8; SD: 2.7). The following factors were significantly more likely for cases than for controls: (a) one to six prenatal care visits (OR: 2.2; 95% CI: 1.16; 4.19; *p* = 0.01); (b) tested for syphilis (not as recommended) only in the second, third trimester, and when hospitalized for giving birth or only one test during pregnancy (OR: 2.24; 95% CI: 1.07; 4.69; *p* = 0.03); and (c) difficulty talking about condom use with their sexual partner (OR: 3.02; 95% CI: 1.27; 7.17; *p* = 0.01).

**Table 2:** Programmatic factors associated with gestational syphilis, in the Brazilian Amazon, 2020–2021.

**Variable**	**Case**	**Control**	**Total**	**Regression**		
	***n* (%)**	***n* (%)**	***n* (%)**	***p***	**OD**	**95% IC**
**Number visits in prenatal care**						
1–6	37 (62.7)	51 (43.2)	88 (49.7)	0.01	2.2	1.16; 4.19
≥7	22 (37.3)	67 (56.8)	89 (50.3)	1		
Mean (SD)	5.8 (2.7)	7.1 (2.7)	6.7 (2.8)			
**Gestational age - prenatal care began (measured in weeks)**						
≤12 weeks	17 (30.9)	50 (42.4)	67 (38.7)	1		
13 to ≥ 28 weeks	38 (69.1)	68 (57.6)	106 (61.3)	0.15	1.64	0.83; 3.23
Did not answer^*^	4	0	4			
Mean (SD)	16.5 (6.6)	14.8 (5.9)	15.3 (6.2)			
**Syphilis screening (Tested for syphilis in the antenatal care or hospital)**						
Not as recommended^**^	47 (79.7)	75 (65.6)	122 (68.9)	0.03	2.24	1.07; 4.69
Recommended^***^	12 (20.3)	43 (36.4)	55 (31.1)	1		
**Difficulty performing rapid tests for syphili**						
No	42 (71.2)	91 (77.1)	133 (75.1)	1		
Yes	17 (28.8)	27 (22.9)	44 (24.9)	0.39	1.36	0.67; 2.77
**Difficulty talking about condom use with sexual partner**						
No	45 (76.3)	107 (90.7)	152 (85.9)	1		
Yes	14 (23.7)	11 (9.3)	25 (14.1)	0.01	3.02	1.27; 7.17
**Received prenatal card in the first visit - antenatal care**						
No	5 (8.5)	9.0 (7.6)	14.0 (7.9)	0.84	1.12	0.35; 3.50
Yes	54 (91.5)	109 (92.4)	163 (92.1)	1		
**Prenatal card in the maternity hospital**						
No	1 (1.7)	5 (4.2)	6 (3.4)	0.39	0.38	0.04; 3.41
Yes	58 (98.3)	113 (95.8)	171 (96.6)	1		
**Sexually transmitted infections test result in the maternity hospital**						
No	11 (18.6)	10 (8.5)	21 (11.9)	0.05	2.47	0.98; 6.21
Yes	48 (81.4)	108 (91.5)	156 (88.1)	1		

* Not considered for statistical calculation.

** Not as recommended: second, third trimester and when hospitalized for giving birth or only one test during pregnancy.

*** Recommended: Tested for syphilis in the first three months, in the third trimester and when hospitalized for giving birth (three time during the pregnancy).

The obstetric and sexual aspect data for the two groups are shown in Table 3. The age at first sexual intercourse was similar among cases (Mean: 16; SD: 2.3) and controls (Mean: 16.5, SD: 2.8). However, gravidity was higher in cases (Mean: 2.5; SD: 1.7) than in controls (Mean: 1.9; SD: 1.3). In regression, a past history of sexually transmitted infections (STI) was approximately 23 times more likely to occur in the case group than in the control group (OR: 22.9; 95% CI: 9.4; 55.8; *p* = 0.00). The cases were more likely to have one to three miscarriages (OR: 4.3; 95% CI: 2.08; 9.16; *p* = 0.00), and multigravida (OR: 2.56; 95% CI: 1.93; 4.90; *p* = 0.00).

**Table 3:** Obstetrics and sexual factors associated with gestational syphilis, in the Brazilian Amazon, 2020–2021.

**Variable**	**Case**	**Control**	**Total**	**Regression**		
	***n* (%)**	***n* (%)**	***n* (%)**	***p***	**OD**	**95% IC**
**Condom use**						
Sometimes	26 (44.0)	57 (48.3)	83 (46.9)	0.34	0.63	0.25; 1.62
Never	23 (39.0)	47 (39.8)	70 (39.5)	0.43	0.68	0.26; 1.77
Always	10 (17.0)	14 (11.9)	24 (13.6)	1		
**Past history of STI**						
No	22 (37.3)	109 (93.2)	131 (74.4)	1		
Yes	37 (62.7)	8 (6.8)	45 (25.6)	0.00	22.9	9.40; 55.8
Did not answer^*^	0	1	1			
**Age at first sexual intercourse (years)**						
≤16	31 (62.0)	64 (55.2)	95 (57.2)	0.41	1.32	0.67; 2.61
17-29	19 (38.0)	50 (44.8)	69 (42.8)	1		
Did not answer^*^	9	2	11			
Mean (SD)	16 (2.3)	16.5 (2.8)	16.3 (2.7)			
**Number of miscarriages**						
None	35 (59.3)	102 (86.4)	137 (77.4)	1		
On to Three	24 (44.7)	16 (13.6)	40 (22.6)	0.00	4.3	2.08; 9.16
**Gravidity**						
Primigravid	20 (33.9)	67 (56.8)	87 (49.1)	1		
Multigravid	39 (66.1)	51 (43.2)	90 (58.9)	0.00	2.56	1.33; 4.90
Mean (SD)	2.5 (1.7)	1.9 (1.3)	2.1 (1.5)			
**Parity**						
Primiparous	27 (45.8)	71 (60.2)	98 (55.4)	1		
Multiparous	32 (54.2)	47 (39.8)	79 (44.6)	0.07	1.79	0.95; 3.36
Mean (SD)	1.9 (1.1)	1.7 (1.2)	1.8 (1.2)			

* Not considered for statistical calculation.

All variables in the binary regressions with *p* < 0.20 were then included in the final multiple logistic regression model (Table 4). The results showed that the case group, compared with the control group, had a lower monthly income, a past history of STI, one to three miscarriages, one to six prenatal care visits, difficulty talking about condom use with their sexual partner, and not receiving a STI test result in the maternity hospital.

**Table 4:** Results of the multiple logistic regression analysis between factors and gestational syphilis, in the Brazilian Amazon, 2020–2021.

**Variable**	***p***	AOR^*^	**95%IC**
**Monthly income (minimal wage)**			
Equal or less than 1 minimum wage	0.04	4.32	1.03; 18.1
Past history of STI^**^			
Yes	0.00	55.4	16.4; 186.6
**Number of miscarriages**			
One to three	0.01	4.34	1.39; 13.5
**Difficulty talking about condom use with sexual partner**			
Yes	0.01	4.92	1.44; 16.7
Received STI^**^test result in the maternity hospital			
No	0.04	4.09	1.06; 15.8
**Number visits in prenatal care**			
One to six visits	0.01	4.93	1.39; 12.03

* Adjusted Odds ratio.

** Sexually transmitted infections.

## Discussion

This case-control study determined the factors associated with GS among postpartum women who were attending PNC programs in the Brazilian Amazon. In present study, the determinants of GS were lower monthly income, a past history of STI, one to three miscarriages, one to six prenatal care visits, difficulty talking about condom use with their sexual partner, and not receiving an STI test result in the maternity hospital.

Brazil has one of the world's largest universal healthcare systems, providing coverage and comprehensive healthcare for women and children in more than 5,500 municipalities (21, 22). The Brazilian Ministry of Health recommends seven or more prenatal care visits during pregnancy and the use of the reverse sequence algorithm for diagnostic syphilis for primary care services of the Unified Health System (22, 23). In 2019, the detection rates of gestational syphilis were 16.5 per 1,000 live births in Pará, a rate below that recorded in the North region (03).

In the present study, all participants used PNC services. However, more than half of the case group reported having only one to six prenatal visits during pregnancy. These women, 69.1% had their first prenatal care visit during the second or third trimester. Consequently, syphilis screening was performed later. The frequency of prenatal care visits was lower than that recommended by the Brazilian Ministry of Health, an important component of quality for PNC (2, 23, 24).

A late first visit is related to the quality of access to PNC (2, 25), including rapid syphilis testing. Early diagnosis makes possible the immediate treatment of pregnant women and their sexual partner (2, 26–28). This result is consistent with the findings of a similar case-control study from Northeast Brazil (8). Another study from Brazil showed that among women attending a prenatal program, one to three PNC visits were associated with nonperformance tests for syphilis (29). Studies conducted in China showed an association between the timing of first treatment for syphilis to adverse pregnancy outcomes and CS (30, 31), evidencing the importance of timing of the first PNC visit. In Brazil, after a positive syphilis reagent test (rapid syphilis test or VDRL), treatment can be provided during the same visit. But this does not exclude the need for a second test for a better diagnostic analysis (12).

The present study showed it was difficult for women to talk to their sexual partners about condom use, as demonstrated in other studies. In PNC visits, health professionals need to involve partners and provide the necessary support to pregnant women (32–34). Adequate treatment of the pregnant woman concomitantly with the treatment of the partner is a determining factor for preventing the vertical transmission of syphilis. To break the chain of transmission, all sexual partners must be educated and treated (4, 22). In Brazil, by the end of 2017, the treatment of the sex partners was taken out from the criteria defining adequate maternal treatment (12).

Studies conducted in Brazil have shown that regional and social inequities, including low income, are factors associated with GS (35, 36). In a population with precarious socioeconomic indicators, primary care professionals play a fundamental role in health education in all areas of care (37, 38). Our study location historically has the lowest percentage of primary care coverage of all the states in the northern region of the Brazilian Amazon (39). This low coverage directly impacts access to prenatal care, the follow-up for pregnant women, and the quality of PNC visits, especially for more vulnerable pregnant women. The CS rate is a consequence of these realities.

The findings of a past history of STI (8–10) and the number of miscarriages (9, 10) were consistent with previous case-control studies among GS cases in Brazil (8), Ethiopia (9) and China (10). Both factors should be investigated by healthcare professionals during prenatal care visits, especially if these diagnoses are associated with syphilis. The diagnosis of syphilis depends on a combination of the reactive test, as well as clinical and epidemiological history, independent of the adopted algorithm for syphilis testing (2, 22). Despite the improvement of public health policies that increase the coverage of primary care and are specific to syphilis, more needs to be done.

This study has some limitations. The GS cases in this study excluded postpartum women who did not attend the PNC program. The study was conducted in only the maternity hospital, and the syphilis status of the women was obtained from medical records, prenatal cards, and secondary data. Further studies in a larger number of maternity hospitals are needed. The findings of the present study include some flaws found in the recruitment of pregnant women, in the link in health responsibility, and in the attributes of the primary care based model. It is necessary to expand the studies to identify the structure and work process continuity in scenarios with a high incidence of gestational and congenital syphilis, as well as to understand the difficulties faced by pregnant women in accessing prenatal care, and to use mixed or qualitative methods to research this problem.

## Conclusion

The study determined that sociodemographic, programmatic, obstetric, and sexual factors are associated with GS among postpartum women who were attending PNC programs in the Brazilian Amazon. The determinants were lower monthly income, a past history of STI, one to three miscarriages, one to six prenatal care visits, difficulty talking about condom use with their sexual partner, and not receiving a STI test result in the maternity hospital.

These results demonstrate that despite the advances in PNC to expand the availability of rapid tests for syphilis and an increase in penicillin prescriptions, there are still gaps in primary care in Pará such as late detected gestational syphilis and inadequate treatment in low-income populations that depend on the public health system. In this context, this study contributes to an analysis of the predictors of the high incidence rate of GS.

## Data availability statement

The original contributions presented in the study are included in the article/Supplementary material, further inquiries can be directed to the corresponding author/s.

## Ethics statement

The studies involving human participants were reviewed and approved by the Research Ethics Committee of the Federal University of Pará under protocol n° 4.134.226. The patients/participants provided their written informed consent to participate in this study.

## Author contributions

TU, EA, RS, EB, and GF conceived and designed the study. GF analyzed the data. TU collected the data. GF, VN, RV, and WA wrote the manuscript. CA-A, GF, and AP wrote the manuscript, provided critical comments, or revised the manuscript. All authors read and approved the final manuscript.

## Funding

This study was supported by the Federal University of Pará (PAPQ-2022).

## Conflict of interest

The authors declare that the research was conducted in the absence of any commercial or financial relationships that could be construed as a potential conflict of interest. The reviewer LM declared a shared affiliation with eight of the authors to the handling editor at the time of review.

## Publisher's note

All claims expressed in this article are solely those of the authors and do not necessarily represent those of their affiliated organizations, or those of the publisher, the editors and the reviewers. Any product that may be evaluated in this article, or claim that may be made by its manufacturer, is not guaranteed or endorsed by the publisher.
