# Supplementary material for: Determinants of gestational syphilis among women attending prenatal care programs in the Brazilian Amazon
Source: Front Public Health. 2022 Nov 9;10:930150. doi: 10.3389/fpubh.2022.930150 (PMC9683036; doi:10.3389/fpubh.2022.930150)
Supplement: Supplementary file 2 [file Data_Sheet_1.docx]

| **TABLE 1** Sociodemographic factors associated with gestational syphilis, in the brazilian Amazon, 2020-2021. | | | | | | | |
| --- | --- | --- | --- | --- | --- | --- | --- |
| **Variable** | **Case** | **Control** | **Total** |  | **Regression** | | |
|  | **n(%)** | **n(%)** | **n(%)** |  | ***p*** | **OR** | **95% IC** |
| **Current age (years)** |  |  |  |  |  |  |  |
| 15-24 | 31(52.5) | 52(44.1) | 83(46.9) |  | 0.64 | 1.30 | 0.44; 3.75 |
| 25-34 | 22 (37.3) | 53 (44.9) | 75(42.4) |  | 0.85 | 0.90 | 0.30; 2.66 |
| 35-41 | 6(10.7) | 13(11) | 19(10.7) |  | 1 |  |  |
| Mean (SD) | 24.8(6.18) | 26.1(6.17) | 25.7(6.18) |  |  |  |  |
| **Education Level** |  |  |  |  |  |  |  |
| Elementary | 17(28.8) | 27(22.9) | 44(24.9) |  | 0.39 | 0.73 | 0.36; 1.48 |
| High school/University | 42(71.2) | 91(77.1) | 133(75.1) |  | 1 |  |  |
| **Marital Status** |  |  |  |  |  |  |  |
| Single | 17 (28.8) | 30(25.4) | 47(26.6) |  | 0.24 | 1.76 | 0.67; 4.59 |
| Stable union | 33(55.9) | 60(50.9) | 93(52.4) |  | 0.22 | 1.71 | 0.72; 4.05 |
| Married | 9(15.3) | 28(23.7) | 37(20.9) |  | 1 |  |  |
| **Monthly income (minimal wage)*** | | | |  |  |  |  |
| Equal or less than 1 minimum wage | 53(89.8) | 78(66.1) | 131(74.0) |  | 0.00 | 4.52 | 1.79; 11.4 |
| More than 1 minimum wage | 6(10.2) | 40(33.9) | 46(26.) |  | 1 |  |  |
| **Current job** |  |  |  |  |  |  |  |
| Housewife/No | 37(62.7) | 52(44.1) | 89(50.3) |  | 0.02 | 2.13 | 1.12; 4.05 |
| Yes | 22(37.3) | 66(55.9) | 88(49.7) |  | 1 |  |  |
| **Access to the internet** | | |  |  |  |  |  |
| No | 2(3.4) | 8(6.8) | 10(5.7) |  | 0.36 | 0.48 | 0.09; 2.34 |
| Yes | 57(96.6) | 110(93.2) | 167(94.3) |  | 1 |  |  |
| Brazilian monthly minimum wage in November 2021 - 1,090 Real per month. | | | | | | | |

| **TABLE 2** Programmatic factors associated with gestational syphilis, in the brazilian Amazon, 2020-2021. | | | | | | | |
| --- | --- | --- | --- | --- | --- | --- | --- |
| **Variable** | **Case** | **Control** | **Total** |  | **Regression** | | |
|  | **n(%)** | **n(%)** | **n(%)** |  | ***p*** | **OD** | **95% IC** |
| **Number visits in prenatal care** | | | |  |  |  |  |
| 1-6 | 37(62.7) | 51(43.2) | 88(49.7) |  | 0.01 | 2.2 | 1.16; 4.19 |
| ≥7 | 22(37.3) | 67(56.8) | 89(50.3) |  | 1 |  |  |
| Mean(SD) | 5.8(2.7) | 7.1(2.7) | 6.7(2.8) |  |  |  |  |
| **Gestational age - prenatal care began (measured in weeks)** | | | | | | | |
| ≤12 weeks | 17(30.9) | 50(42.4) | 67(38.7) |  | 1 |  |  |
| 13 to ≥ 28 weeks | 38(69.1) | 68(57.6) | 106(61.3) |  | 0.15 | 1.64 | 0.83; 3.23 |
| Did not answer* | 4 | 0 | 4 |  |  |  |  |
| Mean(SD) | 16.5(6.6) | 14.8(5.9) | 15.3(6.2) |  |  |  |  |
| **Syphilis screening (Tested for syphilis in the antenatal care or hospital )** | | | | | | | |
| Not as recommended** | 47(79.7) | 75(65.6) | 122(68.9) |  | 0.03 | 2.24 | 1.07; 4.69 |
| Recommended*** | 12(20.3) | 43(36.4) | 55(31.1) |  | 1 |  |  |
| **Difficulty performing rapid tests for syphilis** | | | | | | | |
| No | 42(71.2) | 91(77.1) | 133(75.1) |  | 1 |  |  |
| Yes | 17(28.8) | 27(22.9) | 44(24.9) |  | 0.39 | 1.36 | 0.67; 2.77 |
| **Difficulty talking about condom use with sexual partner.** | | | | | | | |
| No | 45(76.3) | 107(90.7) | 152(85.9) |  | 1 |  |  |
| Yes | 14(23.7) | 11(9.3) | 25(14.1) |  | 0.01 | 3.02 | 1.27; 7.17 |
| **Received prenatal card in the first visit - antenatal care** | | | | | | |  |
| No | 5(8.5) | 9.0(7.6) | 14.0(7.9) |  | 0.84 | 1.12 | 0.35; 3.50 |
| Yes | 54(91.5) | 109(92.4) | 163(92.1) |  | 1 |  |  |
| **Prenatal card in the maternity hospital** | | | | | | | |
| No | 1(1.7) | 5(4.2) | 6(3.4) |  | 0.39 | 0.38 | 0.04; 3.41 |
| Yes | 58(98.3) | 113(95.8) | 171(96.6) |  | 1 |  |  |
| **Sexually transmitted infections test result in the maternity hospital** | | | | | | | |
| No | 11(18.6) | 10(8.5) | 21(11.9) |  | 0.05 | 2.47 | 0.98; 6.21 |
| Yes | 48(81.4) | 108(91.5) | 156(88.1) |  | 1 |  |  |
| *Not considered for statistical calculation. . ** Not as recommended: second, third trimester and when hospitalized for giving birth or only one test during pregnancy  *** Recommended: Tested for syphilis in the first three months, in the third trimester and when hospitalized for giving birth (three time during the pregnancy). | | | | | | | |

| **TABLE 3** Obstetrics and sexual factors associated with gestational syphilis, in the brazilian Amazon, 2020-2021. | | | | | | | |
| --- | --- | --- | --- | --- | --- | --- | --- |
| **Variable** | **Case** | **Control** | **Total** |  | **Regression** | | |
|  | **n(%)** | **n(%)** | **n(%)** |  | ***p*** | **OD** | **95% IC** |
| **Condom use** |  |  |  |  |  |  |  |
| Sometimes | 26(44.0) | 57(48.3) | 83(46.9) |  | 0.34 | 0.63 | 0.25; 1.62 |
| Never | 23(39.0) | 47(39.8) | 70(39.5) |  | 0.43 | 0.68 | 0.26; 1.77 |
| Always | 10(17.0) | 14(11.9) | 24(13.6) |  | 1 |  |  |
| **Past history of STI** | | | |  |  |  |  |
| No | 22(37.3) | 109(93.2) | 131(74.4) |  | 1 |  |  |
| Yes | 37(62.7) | 8(6.8) | 45(25.6) |  | 0.00 | 22.9 | 9.40; 55.8 |
| Did not answer* | 0 | 1 | 1 |  |  |  |  |
| **Age at first sexual intercourse (years)** | | | |  |  |  |  |
| ≤16 | 31(62.0) | 64(55.2) | 95(57.2) |  | 0.41 | 1.32 | 0.67; 2.61 |
| 17-29 | 19(38.0) | 50(44.8) | 69(42.8) |  | 1 |  |  |
| Did not answer* | 9 | 2 | 11 |  |  |  |  |
| Mean(SD) | 16(2.3) | 16.5(2.8) | 16.3(2.7) |  |  |  |  |
| **Number of** miscarriage | | |  |  |  |  |  |
| None | 35(59.3) | 102(86.4) | 137(77.4) |  | 1 |  |  |
| On to Three | 24(44.7) | 16(13.6) | 40(22.6) |  | 0.00 | 4.3 | 2.08; 9.16 |
| **Gravidity** |  |  |  |  |  |  |  |
| Primigravid | 20(33.9) | 67(56.8) | 87(49.1) |  | 1 |  |  |
| Multigravid | 39(66.1) | 51(43.2) | 90(58.9) |  | 0.00 | 2.56 | 1.33; 4.90 |
| Mean(SD) | 2.5(1.7) | 1.9(1.3) | 2.1(1.5) |  |  |  |  |
| **Parity** |  |  |  |  |  |  |  |
| Primiparous | 27(45.8) | 71(60.2) | 98(55.4) |  | 1 |  |  |
| Multiparous | 32(54.2) | 47(39.8) | 79(44.6) |  | 0.07 | 1.79 | 0.95; 3.36 |
| Mean(SD) | 1.9(1.1) | 1.7(1.2) | 1.8(1.2) |  |  |  |  |
| *Not considered for statistical calculation. | | | | | | | |

| **TABLE 4** Results of the multiple logistic regression analysis between factors and gestational syphilis, in the brazilian Amazon, 2020-2021. | | | |
| --- | --- | --- | --- |
| **Variable** | ***p*** | **AOR*** | **95%IC** |
| **Monthly income (minimal wage)** | | | |
| Equal or less than 1 minimum wage | 0.04 | 4.32 | 1.03; 18.1 |
| **Past history of STI**** |  |  |  |
| Yes | 0,00 | 55.4 | 16.4; 186.6 |
| **Number of miscarriage** |  |  |  |
| One to three | 0.01 | 4.34 | 1.39; 13.5 |
| **Difficulty talking about condom use with sexual partner** | | | |
| Yes | 0.01 | 4.92 | 1.44; 16.7 |
| **Received STI** test result in the maternity hospital** | |  |  |
| No | 0.04 | 4.09 | 1.06; 15.8 |
| **Number visits in prenatal care** |  |  |  |
| One to six visits | 0.01 | 4.93 | 1.39; 12.03 |
| *Adjusted Odds ratio. ** Sexually transmitted infections. | | |  |
